# Time-Restricted Eating, ANGPTL4, and Reduction in Residual Cardiovascular Risk

**DOI:** 10.3390/jcm14197026

**Published:** 2025-10-03

**Authors:** Alejandro Gugliucci

**Affiliations:** Glycation, Oxidation and Disease Laboratory, Touro University California, Vallejo, CA 94592, USA; alejandro.gugliucci@gmail.com

**Keywords:** time-restricted eating, intermittent fasting, insulin resistance, liver fat, TRL, ANGPTL, apoCIII, chylomicrons, VLDL, LDL, LPL, atherogenesis, remnants, Lp(a)

## Abstract

Atherosclerotic cardiovascular disease treatment is being reevaluated, since a residual cardiovascular risk (RCR) persists even in patients who achieve optimal LDL-C values. Underlying causes are metabolic dysfunction, lipoprotein(a), inflammation, and triglyceride-rich lipoproteins and their remnants. Dietary treatment options like time-restricted eating (TRE) are becoming more widely acknowledged for their potential advantages in metabolic health and weight control, as a treatment of atherosclerosis expanding beyond LDL-C medication. Beyond weight loss, TRE (which restricts meals to a window of 6 to 8 h) appears as the most accessible treatment, and has been shown to improve blood pressure, lipid profiles, and glucose regulation through mechanisms like metabolic switching and circadian synchronization. We hypothesize, and will present our arguments, that a key mechanism underlying the cardiovascular and weight-related benefits of TRE is its impact on the circadian regulation of angiopoietin-like protein 4 (ANGPTL4) activity within adipose tissue. Additionally, lipolysis is accelerated by ANGPTL4 activation. TRE, via its actions on ANGPTL4, therefore not only inhibits adipose fatty acid uptake but stimulates their release as well. Additionally, TRE may increase intravascular very low-density lipoprotein (VLDL) catabolism by muscle due to the reduced exposure of lipoprotein lipase (LPL) to competing chylomicrons, known to slow the rate of VLDL catabolism. During the prolonged fasting, VLDL residence time is thus shortened, limiting the exposure to endothelium and hepatic lipases and thus reducing the amount of atherogenic remnant particles. Larger, longer-term randomized controlled studies in a variety of groups are required to further clarify TRE’s function in RCR prevention and therapy. As knowledge of triglyceride lipoprotein (TRL) metabolism expands, a comprehensive strategy for the management of RCR emerges, and a broader spectrum of LPL regulator-based therapeutics is created. Consequently, it is advisable to prioritize further research into the influence of TRE on LPL modulation via ANGPTL4 and ANGPTL8, which provides a natural, accessible, and low-cost alternative.

## 1. Introduction

Cardiovascular disease (CVD) persists as the foremost cause of morbidity and mortality across the globe, despite considerable progress in the management of established risk factors [1,2,3,4,5]. A significant issue within contemporary cardiovascular medicine is the ongoing incidence of cardiovascular events—termed “residual cardiovascular risk” (RCR)—that arise even among patients who meet recommended low-density lipoprotein cholesterol (LDL-C) targets [6,7,8,9,10,11,12,13]. This reality necessitates a fundamental reassessment of the prevailing approaches to atherosclerotic cardiovascular disease (ASCVD).

While earlier strategies focused predominantly on lipid reduction, current perspectives recognize the multifactorial nature of RCR. Factors such as inflammation, elevated triglyceride-rich lipoproteins (TRL), lipoprotein(a), and metabolic abnormalities independently drive disease progression [6,9,11,12]. With the growing paradigm shift toward a holistic understanding of atherosclerosis that moves beyond an exclusive focus on LDL-C and pharmacological treatments, it is essential to consider dietary interventions as valuable alternatives.

Time-restricted eating (TRE) represents one such promising dietary intervention that employs circadian rhythms to enhance metabolic health and facilitate weight management residual risk [14,15,16,17]. The advantages of TRE extend beyond mere weight loss; this approach also positively influences glucose homeostasis and insulin kinetics, blood pressure, and lipid profiles through processes such as circadian alignment and metabolic switching [14,18,19,20,21,22]. Consequently, there is considerable scientific interest in TRE as a potential strategy to address RCR. Emerging research indicates that TRE reduces circulating TRL and remnants, which are key atherogenic particles [23,24,25]. The underlying mechanisms we will focus on include the favorable modulation of lipoprotein lipase (LPL) activity, orchestrated by the regulation of ANGPTL 3, 4, and 8 during fasting periods.

This perspective’s main objective is to highlight the necessity for further investigation into the modulation of LPL during TRE. We hypothesize, with supporting evidence, that a principal mechanism underlying the cardiovascular and weight-related benefits of TRE is its influence on the circadian regulation of angiopoietin-like protein 4 (ANGPTL4) activity in adipose tissue. Furthermore, TRE (reducing the exposure of lipoprotein lipase—LPL—to competing chylomicrons) further optimizes very low-density lipoprotein (VLDL) catabolism in muscle tissue.

Following a summary of recent progress in residual cardiovascular risk research, we focus on TRE, examining its impact on the ANGPTL3/4 axis and emphasizing the central role of ANGPTL8. We suggest that a deeper insight into how TRE influences LPL may lead to advances capable of alleviating the considerable RCR present after LDL-C optimization and potentially reducing the reliance on expensive (and in several cases yet not fully studied) emerging interventions such as anti-inflammatory agents, selective lipoprotein (a) [Lp(a)]-lowering therapies, and cardio-protective metabolic medications. Lifestyle interventions, including consideration of TRE as we shall discuss, could prove essential for achieving optimal patient outcomes and cardiovascular risk management. Consequently, the key message presented here is that, while ongoing research is vital to confirm the efficacy of emerging and combination therapies addressing inflammation, Lp(a), and metabolic dysfunction, it is equally important to assess the integration of these treatments alongside TRE modalities. This combined approach may offer further advantages in reducing RCR.

## 2. Residual Cardiovascular Risk

Residual cardiovascular risk describes the continued occurrence of ASCVD events—including stroke, myocardial infarction, and cardiovascular death—in individuals who have already attained guideline-recommended control of traditional risk factors, particularly LDL-C [6,7,8,9,10,11,13]. Though lipid-lowering therapies, especially statins, have profoundly improved cardiovascular outcomes, a notable portion of patients remain vulnerable to recurrent events. This enduring risk exposes limitations in current, widely accepted treatment paradigms and prompts a critical reflection regarding what truly constitutes “optimal” cardiovascular care.

### 2.1. Main Pathophysiological Mechanisms Explaining RCR

The concept of residual risk challenges the sufficiency of achieving LDL-C targets alone. More than a theoretical concern, this issue has significant clinical implications, highlighting the complexity of atherosclerosis and the necessity of addressing additional interrelated disease pathways beyond dyslipidemia. Accordingly, effective management of residual risk requires the investigation and intervention of non-LDL-C mechanisms that contribute to cardiovascular pathology [6,7,8,10,11].

**Figure 1:**
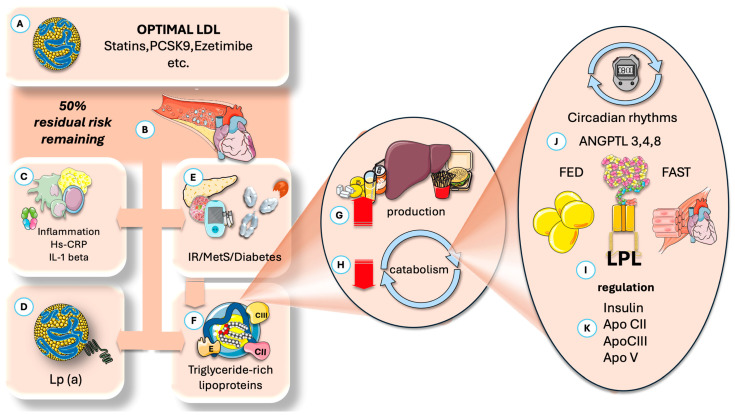
Residual cardiovascular risk, importance, and main mechanisms proposed. (**A**) Residual risk is the remaining risk after optimization of (**B**) LDL-C levels. (**C**) Inflammation plays a key role. (**D**) High levels of Lp (a) prevalent in 10% of the population. (**E**) Subjacent insulin resistance and its expression as MetS or diabetes are important factors. (**F**) Increased TRL and its remnants are receiving much attention and are the focus of this article. Increased TRL may be due to either (**G**) increased production (fatty acids, fructose, alcohol) or decreased catabolism (**H**), or both. Catabolism, in turn, depends on LPL and its complex regulation. It has a key circadian component (**J**), where ANGPTL proteins play a critical role in cycling fast and fed status in different tissues, of which ANGPTL4 and 8 are the focus of this paper. (**I**) LPL regulation also depends on optimal insulin action and the role of activators and inhibitors, of which apoCIII has the most attention at present (**K**). ANGPTL: angiopoietin-like protein; Lp(a): lipoprotein (a); IR: insulin resistance; MetS: metabolic syndrome; TRL: triglyceride-rich lipoproteins; LPL: lipoprotein lipase. The Figure was partly generated using Servier Medical Art, provided by Servier, licensed under a Creative Commons Attribution 3.0 unported license.

Figure 1 presents a summary of the current knowledge regarding the main underlying factors of RCR, which may amount to up to 50% of total risk. Statins remain central for lowering LDL-C and reducing cardiovascular events, with agents like ezetimibe and proprotein convertase subtilisin/kexin type 9 (PCSK9) inhibitors offering further benefits. However, considerable residual risk remains due to at least four major factors beyond LDL-C: inflammation, elevated Lp(a), elevated TRL, and their remnants, as well as metabolic dysfunction.

#### 2.1.1. Inflammation

Inflammation contributes to residual cardiovascular risk by promoting atherosclerotic plaque formation, progression, instability, and rupture. Inflammatory cells in arteries release cytokines that impair the endothelium, increase LDL-C accumulation, and destabilize plaques, raising the risk of heart attacks and strokes. Inflammation also drives vascular calcification and post-myocardial infarction remodeling, exacerbating heart failure. Systemic inflammation is linked to insulin resistance and chronic kidney disease, further elevating risk [26,27,28,29,30,31,32].

Inflammation, now recognized as a major cause of atherosclerosis progression, can be monitored with high-sensitivity C-reactive protein (hsCRP). Elevated hsCRP identifies increased residual risk, as persistent inflammation promotes plaque instability and thrombotic events [33,34,35,36]. The CANTOS trial showed that targeting inflammation with canakinumab, an anti-interleukin-1β (IL-1 β) antibody, reduced cardiovascular incidents in patients with prior heart attacks and high hsCRP, independent of lipid lowering [35,36,37,38,39]. This underlines the importance of managing inflammation alongside lipids for effective risk reduction. Identifying precise inflammatory pathways like IL-1β allows for more targeted interventions, supporting a shift toward precision medicine and personalized therapy using biomarkers such as hsCRP [29,30,31]. The JUPITER trial showed statins reduced events in people with elevated hsCRP. Colchicine trials (COLCOT, LoDoCo2) confirmed the benefits, while the CIRT trial found methotrexate ineffective, indicating only certain discrete inflammatory pathways are relevant [26,27,32].

#### 2.1.2. Lipoprotein(a) [Lp(a)]

Lp(a) is a genetically determined, independent risk factor for ASCVD, myocardial infarction, and aortic valve stenosis. A significant challenge with Lp(a) is that its levels are largely unaffected by conventional lipid-lowering therapies such as statins, ezetimibe, or PCSK9 inhibitors, making it a key component of the persistent residual risk [40,41,42]. Lp(a) exerts its atherogenic effects through multiple mechanisms, including pro-inflammatory, pro-thrombotic, and pro-oxidative properties, which contribute to its role in plaque development and instability [40,42,43,44,45]. The fact that Lp(a) is an independent and genetically determined risk factor, largely unaffected by existing therapies, means that even if a patient’s LDL-C is at goal, these other lipid components can still drive disease. This implies that a standard lipid panel might be insufficient for complete risk assessment, justifying intense research into novel therapies designed to lower Lp(a). Innovations in lowering Lp(a), such as antisense oligonucleotides and small interfering RNAs (siRNAs), are being tested and may enable targeted, long-acting treatments [11].

#### 2.1.3. Insulin Resistance (IR)

Insulin resistance (IR) and the ensuing metabolic dysfunction are complex: simply controlling glucose or weight often does not fully reduce ASCVD risk. IR, per se, has a key impact on TRL metabolism, as we shall delve into later; therefore, it remains a significant factor to be addressed and controlled [46]. Sodium-glucose co-transporter-2 (SGLT-2) inhibitors and Glucagon-like peptide-1 (GLP-1) receptor agonists, as well as dual action new peptides, address metabolic risks, offering benefits beyond glucose control [47,48,49,50]. Additional clinical contributors to residual risk include chronic kidney disease, sleep apnea, psychosocial stress, and genetics. New biomarkers are under study to improve risk prediction and guide tailored therapies.

#### 2.1.4. Triglyceride-Rich Lipoproteins and Remnants

**Figure 2:**
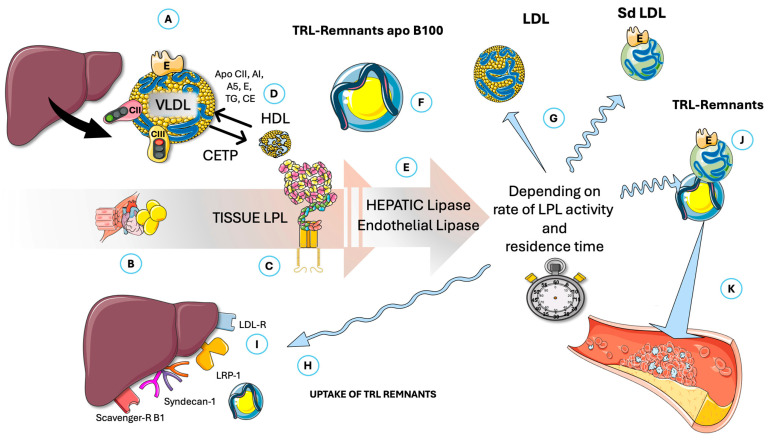
Overview of fasting TRL intravascular catabolism and potential production of atherogenic remnants. (**A**) Liver produces VLDL, which is catabolized mainly by (**B**) muscle in the fasting state. VLDL carries (among others) apoB100, apoE (critical for reuptake of its remnants), apo CII, and apoCIII (activator and inhibitor or LPL, respectively). (**C**) Lipoprotein lipase (LPL) releases FA for tissue oxidation, thereby reducing VLDL size. The whole process is regulated by activators and exchanges of lipid and apolipoproteins with HDL. (**D**). The importance of cholesteryl-ester transfer protein (CETP) is underlined. Smaller size VLDLs are then acted upon by hepatic and endothelial lipases (**E**) in a delicate balance that produces LDL or small–dense LDL (**G**) and, depending on residence time (balanced or imbalanced actions of apoE, CII, and CIII), may produce atherogenic TRL remnants (**F** and **J**) that are either captured by the liver (**H**) through several specific receptors (**I**) or may linger to enter subintima in arteries and result in atherogenesis (**K**). CII: apoCII; CIII: apoCIII; E: apoE; CETP: cholesteryl-ester transfer protein; sd-LDL: small–dense LDL; LDL-R: LDL receptor; LRP-1: LDL-receptor like protein 1; VLDL: very low-density lipoproteins. The Figure was partly generated using Servier Medical Art, provided by Servier, licensed under a Creative Commons Attribution 3.0 unported license.

Figure 2 summarizes VLDL metabolism (precisely the most relevant TRL for our discussion on fasting), showing how liver production, secretion, and remnant uptake—balanced against catabolism by capillary LPL and other lipases—influence remnant entry into artery walls, where they contribute to atherogenesis. This article concentrates on VLDL catabolism and therefore examines the regulation of lipoprotein lipase (LPL) and the influence of fasting on this process. Despite optimal LDL-C levels, elevated TRLs, which lead to high serum triglycerides (TG) and remnant cholesterol, are significant contributors to RCR [51,52,53,54,55,56,57]. These TRLs are very sensitive to IR; indeed, they make serum triglycerides (TG) and their indexes (such as TG/HDL-C, TG/glucose, etc.) convenient markers both of IR and CVD risk. TRL remnants are highly atherogenic (several times more than LDL when measured by their relative cholesterol contents), promoting inflammation and endothelial dysfunction, thereby exacerbating plaque formation and progression [55,56,58]. This necessitates the development of therapies targeting these specific non-LDL-C pathways, be they pharmacological, as we shall discuss, or dietary interventions, which are our focus here.

As previously briefly introduced in Figure 1, LPL activity is regulated in fed and fasted states by specific angiopoietin-like proteins (ANGPTL): ANGPTL3, ANGPTL4, and ANGPTL8, with the latter serving as a principal modulator. Accordingly, the subsequent sections will review the key aspects of time-restricted eating (TRE), followed by an overview of LPL and its primary regulators. We propose, and we will articulate the supporting arguments, that one of the central mechanisms underlying the cardiovascular and weight-related benefits of TRE involves its effects on the circadian rhythm of ANGPTL4 activity in adipose tissue, as well as its optimization of VLDL catabolism by muscle, potentially potentiated by reduced ANGPTL8 levels [51,52,53,54,55,56,57,58].

## 3. Time-Restricted Eating Represents One of the Most Feasible and Accessible Approaches to Intermittent Fasting

Intermittent Fasting (IF) serves as a broad umbrella term encompassing various eating patterns characterized by alternating periods of voluntary food abstinence and eating. A fasting period is generally considered to be at least 12 h [59,60,61,62,63,64]. Several modalities exist, and it must be noted that each approach impacts health differently [65,66,67].

### 3.1. Intermittent Fasting Modalities

∗ Time-restricted eating/fasting (TRE/TRF): This approach confines daily caloric intake to a defined window within each 24 h period. The most widely researched protocol is the 16 h fasting period, followed by an 8 h eating window, commonly known as the 16:8 method. This modality serves as the core element of our discussion.
∗ Alternate-Day Fasting (ADF) involves alternating between days of normal, unrestricted food consumption and days characterized by either complete fasting or substantial calorie reduction (for example, approximately 500 calories).
∗ 5:2 Intermittent Fasting: Individuals eat normally five days per week, with calorie intake significantly restricted (typically 0–25% of estimated energy needs, or roughly 500–600 calories) on two non-consecutive or consecutive “fasting” days.
∗ Prolonged fasting: This refers to abstaining from caloric intake for periods extending 24 h or more, differing fundamentally from the daily time-restricted approach.

The observation that TRE is generally better tolerated and has lower dropout rates suggests that TRE might be a more practical and sustainable choice for a broader population [23,24,68,69,70,71,72]. This underscores that the most effective approach is often the one that can be consistently adhered to safely. From this perspective, we concentrate only in TRE.

### 3.2. Health Effects of TRE: Overall Benefits

Studies in animals and humans indicate that TRE can lower risks of metabolic diseases, cancer, and neurodegenerative conditions, while slowing aging. TRE has been shown to improve insulin sensitivity, blood pressure, body composition, and lipid metabolism [16,21,22,73,74,75]. It supports metabolic and organ health, aids tissue regeneration, and may increase the efficacy of chemotherapy, likely through circadian rhythm synchronization. TRE also influences the gut microbiome, enhance autophagy and mitochondrial function, and supports immune responses against tumors [76,77,78,79].

### 3.3. Specific Benefits of TRE

Clinical trials assessing TRE typically utilize daily eating windows ranging from 6 to 10 h. An 8 h eating window (e.g., from 10:00 to 18:00 h, also known as early TRE) has been frequently studied and consistently shown to be effective for both weight loss and fat loss [23,68]. A very short 4-h eating window did not demonstrate additional health benefits compared to longer windows and was associated with an increase in minor adverse events such as headaches, moodiness, and nausea. Conversely, a longer 12 h eating window generally did not yield significant health benefits. This data clearly indicates an optimal “sweet spot” for the eating window duration (6–8 h). Both excessively short (4 h) and overly long (12 h) windows are shown to be less effective. This collective data points to an optimal time window, and going too short or too long can negate benefits or even introduce risks. This is a vital practical implication for anyone considering TRE, moving beyond a simplistic “less is more” mindset [23,68,69,71,80].

#### 3.3.1. Cardiometabolic Health Improvements

Research on TRE has demonstrated several short-term benefits with respect to cardiovascular health [20,21,22,23,25,73,74,75,81]:

- Blood pressure improvement: Multiple studies indicate that TRE may contribute to reduced blood pressure, thereby mitigating a significant cardiovascular risk factor [76,78].
- Cardiometabolic lipoproteins: Evidence from several studies suggests that TRE can improve LDL-cholesterol and TRL levels; the effects on HDL-C remain inconclusive [20,21,22,23,25,73,74,75,81].
- Enhanced insulin sensitivity: TRE has the potential to increase insulin responsiveness and lower blood glucose levels, which may reduce the risk of type 2 diabetes—a key risk factor for heart disease [76].
- Inflammation reduction: Some research supports that TRE may alleviate chronic inflammation, a condition linked to the progression of atherosclerosis [35,37].

These findings highlight TRE as a promising approach for supporting cardiovascular health, though further investigation is warranted to understand its long-term effects. Notably, these benefits can occur independently of weight loss or even without weight loss, particularly in individuals with prediabetes or metabolic syndrome. The consistent finding that TRE improves health markers without weight loss and specifically benefits populations with existing conditions, like prediabetes or metabolic syndrome, suggests its utility as a therapeutic intervention for managing chronic metabolic diseases (RCR included) rather than solely a preventative lifestyle choice for healthy individuals. If TRE’s benefits were exclusively tied to weight loss, its role would be limited to weight management [20,21,22,23,25,73,74,75,81]. However, the explicit mention of improvements without weight loss and its demonstrated efficacy in individuals already experiencing metabolic dysfunction points to a direct, independent physiological impact on metabolic pathways. This renders it even more suitable to address RCR, as we will delve into. What are the mechanisms involved?

**Figure 3:**
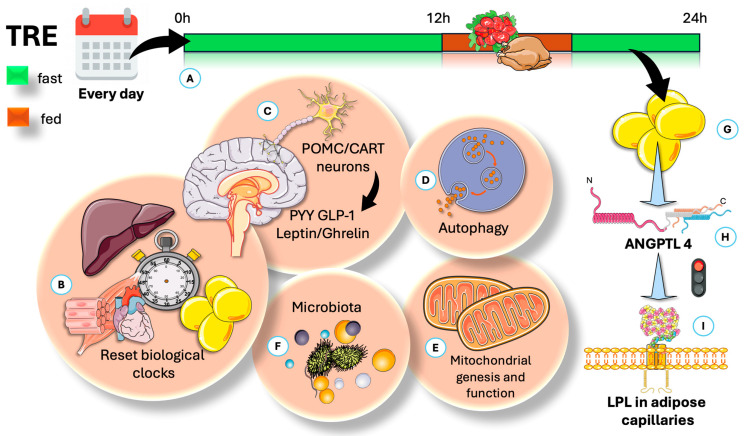
Time-restricted eating (TRE) is a dietary strategy associated with several potential benefits for weight management and cardiovascular health. TRE produces health benefits through five known pathways, along with an additional mechanism proposed in this article. (**A**) This method restricts daily calories to a set time frame. The most studied is the 16:8 protocol—16 h fasting, 8 h eating. (**B**) Circadian rhythms, governed by molecular clocks, help cells and organisms adjust to routine environmental changes like activity cycles and energy intake. Overall, TRE can help reset the circadian clock by affecting AgRP-regulated circadian genes and raising the liver AMP/ATP ratio. (**C**) Circadian clocks include the master pacemaker in the suprachiasmatic nucleus (SCN) of the hypothalamus. The hypothalamus controls appetite through anti-appetite neurons (POMC, CART) and appetite-stimulating neurons (NPY, AgRP). In addition, TRE influences appetite by affecting hormones and peptides such as leptin, insulin, and ghrelin. (**D**) TRE promotes repairing autophagy by increasing AMPK and reducing mTORC activities. (**E**) By the same mechanism, it increases mitochondrial function and may reduce inflammation. (**F**) TRE may affect the rhythm of intestinal microbiota by promoting the periodic attachment of segmented filamentous bacteria to epithelial cells. (**G**) We propose that an important effect of TRE is persistent secretion of ANGPTL4 (**H**), which shifts fat to muscles by inhibiting LPL in adipose capillaries (**I**), as further illustrated in Figure 4. AgRP: agouti-related neuropeptide; AMPK: AMP-regulated kinase; mTORC: mammalial target of rapamycin C; ANGPTL4: angiopoietin-like protein 4; NPY: neuropeptide Y; POMC: pro-opium melanocortin; CART: cocaine–amphetamine-regulated transcript. The Figure was partly generated using Servier Medical Art, provided by Servier, licensed under a Creative Commons Attribution 3.0 unported license.

Figure 3 provides an overview of TRE, highlighting the most effective time modality for promoting compliance (early fasting, skipping breakfast), as well as the five established mechanisms traditionally cited to account for its positive impact. Additionally, we introduce a sixth mechanism that warrants further investigation and scholarly attention.

TRE offers benefits through several physiological mechanisms: aligning eating times with circadian rhythms, inducing metabolic switching, and often reducing caloric intake. Irregular meal timing can disrupt peripheral organ clocks, even when calorie intake remains unchanged, leading to negative metabolic effects. TRE addresses this by restricting food intake to the active part of the day, re-synchronizing internal clocks and supporting better metabolism.

Metabolic switching occurs during daily fasting periods, better if they are (16–18 h), shifting energy use from glucose to fats and ketones. This transition supports cellular repair by autophagy, increases mitochondrial function, and may reduce inflammation. Fasting also influences genes and proteins related to circadian regulation, such as (among others) AMP activated kinase (AMPK), mammalian target of rapamycin (mTOR), and SIRT1 [23,69,71]. Restricting the eating window typically leads to an unintentional calorie reduction—mainly by cutting out late-night snacks—and studies show TRE participants often consume fewer calories effortlessly. Importantly, earlier meal timing can improve weight loss outcomes compared to eating later in the day. Overall, the health benefits of TRE emerge from a combination of improved metabolic timing, enhanced fasting-induced adaptations, and lower calorie intake, suggesting its advantages go beyond simple calorie restriction. Given that this perspective centers on lipid metabolism and RCR (Section 4, Section 5, Section 6 and Section 7), only a summary of information beyond lipid metabolism will be provided in Section 3 below. Readers are encouraged to consult comprehensive reviews available in the literature for further details on related topics [17,73,74,75].

#### 3.3.2. Effect on Circadian Rhythms

Circadian rhythms are maintained by molecular clocks and function as internal timing mechanisms, allowing cells, organs, and organisms to adapt to regular environmental changes such as activity-rest cycles and energy intake [17,82,83,84].

Circadian clocks include the master pacemaker in the suprachiasmatic nucleus (SCN) of the hypothalamus and secondary clocks in peripheral organs like the liver, heart, muscle, and adipose tissue [82,84].

The hepatic biological clock can be adjusted by mealtime. This process involves the rhythmic transcription of thousands of genes that participate in various physiological activities, including the agouti-related neuropeptide (AgRP) and AMPK.

The available evidence indicates that TRE interacts with liver rhythms by influencing the expression of several rhythmic regulators. TRE has been shown to quickly increase AgRP expression. AgRP is involved in upregulating hepatic rhythm-associated transcription factors [17,83,84].

TRE activates the fasting-sensitive protein kinase AMPK through increased AMP levels. AMPK influences the circadian clock by regulating key circadian clock components.

Overall, TRE can help reset the circadian clock by affecting AgRP-regulated circadian genes and raising the liver AMP/ATP ratio [17,82,83,84,85,86,87,88,89].

TRE may influence circadian rhythms in muscle, white adipose tissue (WAT), gut microbiota, and other organs [17,82,83,84].

In addition, studies indicate that TRE can influence the circadian rhythms of various regulators involved in adipose tissue function, such as fibroblast growth factor 21 (FGF21) and hormone-sensitive lipase (HSL) [83,84,90,91,92]. We will address the importance of HSL activation by ANGPTL4 later.

#### 3.3.3. Microbiota

Several studies examined the relationship between TRE, gut microbiota, and metabolic health. The results indicate that late TRE combined with energy may not maintain or enhance gut microbiota diversity as effectively as early TRE with energy restriction or energy restriction alone. Furthermore, TRE was associated with potentially greater long-term benefits and an increase in gut microbes related to metabolic health—such as Faecalibacterium and Subdoligranulum, which might be linked to reductions in fasting glucose and diastolic blood pressure. Associations observed were modest, unadjusted for confounders, and do not establish causality. Between-group analyses at the conclusion of the intervention and during follow-up showed no significant changes at the phylum level or in beta diversity [93,94,95,96,97,98,99].

Additionally, TRE may be linked to the rhythmicity of intestinal microbiota [93,97,100]. TRE has been shown to induce the rhythmic attachment of segmented filamentous bacteria to intestinal epithelial cells. This attachment results in diurnal patterns in the expression and activation of signal transducers, which regulate immune responses and influence the rhythms of antimicrobial proteins, thereby promoting intestinal innate immunity. Other research indicates that TRE can modulate gut microbiota composition, though its relationship with circadian rhythms remains unclear. Additional studies are thus required to further investigate the mechanisms behind these associations and explore the therapeutic potential of personalized TRE strategies for CVD and/or obesity management. Future research should also consider microbial populations in the upper gastrointestinal tract and possible intestinal tissue remodeling to improve the understanding of the gut microbiome’s role in metabolic regulation.

#### 3.3.4. Appetite

Also illustrated in Figure 3, TRE has been demonstrated to significantly influence the regulation of appetite [62,80]. The hypothalamus serves as the central control center for appetite, with anti-appetite neurons—specifically pro-opiomelanocortin (POMC) and cocaine–amphetamine-regulated transcript (CART) neurons—and appetite-stimulating neurons, such as neuropeptide Y (NPY) and agouti-related peptide (AgRP) neurons, playing key regulatory roles [62,77,101]. Furthermore, TRE can regulate appetite through various hormones and peptides, including leptin, insulin, and ghrelin. Leptin and insulin regulate food intake by activating POMC/CART neurons and inhibiting NPY/AgRP neurons in the arcuate nucleus [62,80,101,102]. In contrast, ghrelin is a hormone that suppresses POMC/CART neurons and stimulates NPY/AgRP neurons.

#### 3.3.5. Metabolism, Insulin

Overall, TRE may enhance glucose and lipid metabolism by synchronizing the circadian clock and regulating rhythmic gene expression. Fasting influences gene expression related to glucose and lipid metabolism through factors like insulin-like growth factor-1 (IGF-1), extracellular-signal regulated kinase (ERK), forkhead box transcription factors (FOXO), and peroxisome proliferator-activated receptors (PPAR) [77,78,101]. Liver-regulated hepatic clocks tightly control circulating IGF-1 [70,103,104]. TRE lowers IGF-1 and insulin during fasting and restores them upon refeeding, helping reset the circadian clock.

Collectively, these recent findings suggest that TRE may offer promising benefits for health improvement. However, there is limited understanding regarding the direct interaction between TRE and the master circadian clock, which warrants further investigation.

#### 3.3.6. Other Effects

There is a paucity of studies on genetics and TRE, which will certainly open avenues for future research. Investigations on the impact of TRE on microRNA have surfaced recently but are scarce, and more depth will be needed to offer a perspective on this interesting topic [21,24,68,71,78,103,104,105,106,107,108,109,110].

## 4. TRL Metabolism: The Pivotal Role of Lipoprotein Lipase in Determining the Fate of Triglyceride-Rich Lipoproteins

**Figure 4:**
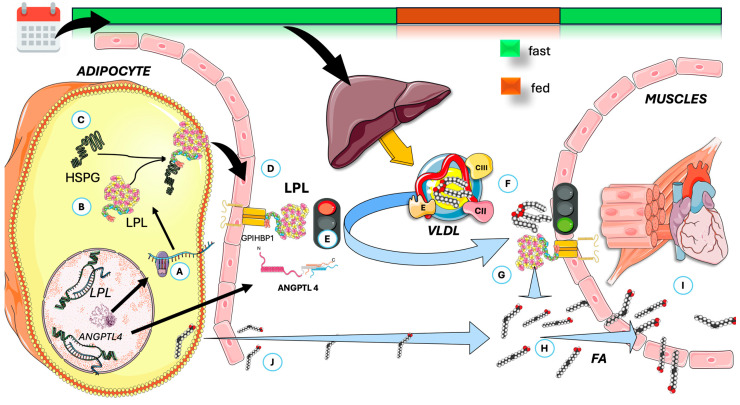
Overview of LPL physiology and the effect of prolonged daily fasting as in TRE on VLDL fate in circulation. (**A**) LPL is synthesized (**B**) and matured in the ER, then (**C**) binds to heparan sulfate proteoglycans (HSPG) during transport, transported to (**D**) capillaries via proteins like GPIHBP1 which also help anchor it to endothelial cells. (**E**) In adipose tissue, fasting induces the production of ANGPTL4, which inhibits LPL. (**F**) The VLDL secreted by the liver is the acted upon, preferentially, by muscle LPL (**G**), which is very active (via other mechanisms). (**H**) It releases FA for (**I**) oxidation in muscle, together with (**J**) those released from adipocyte stores. ANGPTL4: angiopoietin-like protein 4; GPIHBP1: Glycosylphosphatidylinositol-anchored high-density lipoprotein binding protein 1; FA: fatty acids; LPL: lipoprotein lipase. The Figure was partly generated using Servier Medical Art, provided by Servier, licensed under a Creative Commons Attribution 3.0 unported license.

In Figure 4, we highlight some key features of lipoprotein lipase (LPL). LPL breaks down triglycerides in circulating TRL (VLDL all day and chylomicrons (CM) in the postprandial period), releasing fatty acids for storage or energy. LPL is produced by macrophages, adipose tissue, muscles, and the brain—not the liver—and functions at the endothelial surface of capillaries [10,56,111,112]. Its production and activity are tightly regulated by various modulators, including insulin, which affects LPL levels in adipocytes. As shown in the Figure, LPL is synthesized and matured in the ER and is then transported to capillaries via Glycosylphosphatidylinositol-anchored high-density lipoprotein binding protein 1 (GPIHBP1), which also helps anchor it to endothelial cells. LPL binds to heparan sulfate proteoglycans (HSPG) during transport, and recent studies show that LPL remains active when complexed with GPIHBP1, not just as a homodimer [10,56,112,113,114,115,116,117,118,119,120,121].

### 4.1. The Function of ANGPTL4 in Regulating LPL Activity During Fed and Fasted States

**Figure 5:**
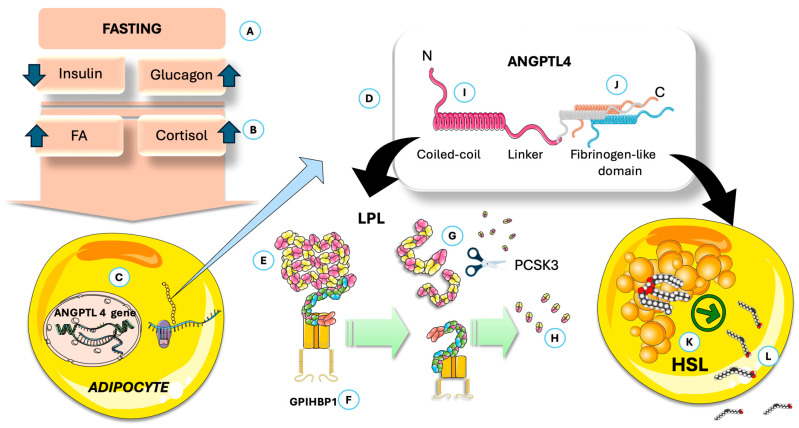
ANGPTL 4 impairs adipose uptake of FA and increases their release from the stores. (**A**) Fasting via its (**B**) hormonal and metabolic milieu, induces ANGPTL4 expression by adipocytes (**C**). (**D**) ANGPTL 4 schematic structure. By its action on LPL, (**E**) it promotes dissociation from GPIHBP1 (**F**); this dissociation (**G**) facilitates the action of PCSK3 (**H**) and proteolysis. These actions depend on an intact C-terminal (**I**). The fibrinogen-like domain (**J**) activates adipocyte HSL (**K**) promoting release of FA (**L**). ANGPTL4: angiopoietin-like protein 4; GPIHBP1: Glycosylphosphatidylinositol-anchored high-density lipoprotein binding protein 1; HSL: hormone-sensitive lipase; LPL: lipoprotein lipase; PCSK3: proprotein convertase subtilisin/kexin type 3. The Figure was partly generated using Servier Medical Art, provided by Servier, licensed under a Creative Commons Attribution 3.0 unported license.

LPL regulates postprandial TG storage and is stimulated by insulin. Its activity in white adipose tissue is high when fed and decreases after fasting, primarily through posttranslational mechanisms, since *LPL* mRNA levels remain largely unchanged between states [117,118,122]. As shown in Figure 4 in a schematic way, VLDL is then poorly metabolized by adipose LPL, which favors muscle metabolism. Fasting increases LPL degradation in the Golgi/post-Golgi compartments and converts active to inactive LPL, a process reliant on activation of a gene other than *LPL*. This gene has been identified as *ANGPTL4*. Studies in genetically modified mice show that ANGPTL4 is essential for regulating LPL [107,108,123,124,125,126,127]. As shown in Figure 5, fasting but not feeding states, overexpressing ANGPTL4, raise TG by reducing their clearance and adipose uptake, while whole-body or partial inactivation lowers plasma TG, boosts adipose LPL activity, facilitates TG clearance, and increases fatty acid uptake.

Figure 5 depicts the simplified structure of ANGPTL4: both the N- and the C-terminal domains have specific discrete actions on adipocytes [108,123,128]. The adipocyte-specific loss of ANGPTL4 highlights its inhibitory effect on adipose LPL and TG clearance. Figure 5 also illustrates that ANGPTL4 facilitates LPL degradation through proprotein convertase subtilisin/kexin type 9 (PCSK3)-mediated cleavage, where the process is initiated by ANGPTL4-induced LPL unfolding. Recent studies show that ANGPTL4 binds near LPL’s catalytic pocket and promotes irreversible unfolding of its hydrolase domain, making LPL more susceptible to PCSK3 cleavage [108,123,129]. This action is mediated by N-terminal His46, Gln50, and Gln53 in ANGPTL4. Recent studies indicate that, as depicted in the figure, the C-terminal domain is involved in the direct activation of hormone-sensitive lipase (HSL). Consequently, under the influence of ANGPTL4, LPL activity in adipocytes is inhibited, thereby reducing fatty acid uptake [107,108,124,128,129,130,131,132]. In addition, ANGPTL4 enhances the release of fatty acids through the stimulation of hormone-sensitive lipase (HSL), a process further amplified by the increased HSL activity associated with low insulin levels. In adipocytes, ANGPTL4 boosts fatty acid release by elevating cAMP levels via its C-terminal fibrinogen-like domain. During fasting, both domains of ANGPTL4 promote the transition from TG storage to mobilization in adipose tissue. Studies in humans show that prolonged fasting reduces LPL activity and mass in adipose tissue, accompanied by increased ANGPTL4 mRNA and protein levels. ANGPTL4 and LPL protein levels are negatively correlated in human adipose tissue. During fasting, both mouse and human adipose tissue exhibit higher ANGPTL4 expression, likely due to reduced insulin, increased glucocorticoids, and elevated free fatty acids, as we depict in Figure 4. Genetic evidence also shows that a truncation variant of ANGPTL4 is linked to lower plasma TG after at least 4 h of fasting [108,129,131,132,133].

Given these considerations, we suggest that the dual and synergistic mechanism illustrated in Figure 4 represents a significant factor contributing to the favorable impact of TRE on weight reduction (through reduced uptake of new fatty acids and depletion of stored fatty acids in adipocytes). This process facilitates the redirection of TRL to muscle tissue for energy utilization, thereby shortening their half-life and preventing remnant build-up, which may lead to atherogenesis. The observed effects may be attributable to the 16+ h window during which these actions take place. A third mechanism, which will be detailed subsequently, completes our conceptual framework.

In summary, ANGPTL4 functions as an autocrine modulator within white adipose tissue during periods of fasting by facilitating the degradation of LPL and reducing the amount of active endothelial LPL responsible for triglyceride hydrolysis. Moreover, the overall effect is that ANGPTL4 activity promotes a reduction in adipose tissue mass. This is significant, as it provides a mechanistic explanation for the efficacy of TRE; by extending ANGPTL4 activity, TRE enhances the outcomes described above.

### 4.2. Antagonistic Regulation of ANGPTL4 Function by ANGPTL8

**Figure 6:**
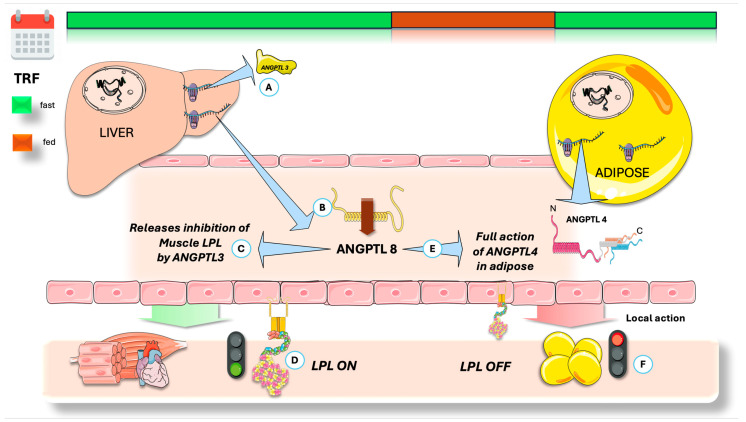
Integration of regulators of the fast-fed cycle of tissue-specific LPL activity: fasting state. (**A**) The liver secretes an inhibitor of muscle LPL, ANGPTL3, keeping a stable concentration throughout the day. ANGPTL8 is needed for full activation of ANGPTL3, by forming a 3/1 ANGPTL3/8 complex that acts on muscle LPL. As seen before, ANGPTL8 secretion is depressed during the fasting state (**B**), reducing the concentration of ANGPTL3/8 complex and (**C**) liberating muscle LPL from inhibition. (**D**) ANGPTL8 is also a potent inhibitor of ANGPTL4. They share structural similarities in their N-terminal region; ANGPTL8 lacks the fibrinogen-like domain. Lower ANGPTL8 during fasting (**E**) allows new ANGPTL4 to work uninhibited, (**F**) shutting down adipose LPL. ANGPTL4: angiopoietin-like protein 4; ANGPTL3: angiopoietin-like protein 3; ANGPTL8: angiopoietin-like protein 8; LPL: lipoprotein lipase. The Figure was partly generated using Servier Medical Art, provided by Servier, licensed under a Creative Commons Attribution 3.0 unported license.

As shown in Figure 6, ANGPTL8 locally counteracts ANGPTL4′s inhibition of LPL by forming a protein complex with it, resulting in a weaker LPL inhibition. This ANGPTL4/8 complex may protect LPL from ANGPTL3/8 inhibition, and ANGPTL8 binding to ANGPTL4 can promote its degradation in adipocytes [107,109,110,133,134,135,136,137,138,139,140,141,142,143]. Overall, ANGPTL8′s inhibitory effect on ANGPTL4 enhances adipose LPL activity after feeding, supporting TG uptake into adipose tissue instead of muscle. Fasting strongly reduces ANGPTL8 expression, thereby releasing all prior inhibition.

### 4.3. Physiological Role of Circulating ANGPTL4

ANGPTL4 is present in human plasma, as detected by different ELISA methods and Western blot. Most plasma ANGPTL4 exists as the C-terminal fragment, which does not inhibit LPL, unlike the full-length or N-terminal forms. Since ANGPTL8 binding inhibits ANGPTL4′s ability to regulate LPL, and the C-terminal fragment cannot inhibit LPL, the current evidence suggests that circulating ANGPTL4 may not play a significant role in LPL regulation. Plasma C-terminal ANGPTL4 rises when non-esterified fatty acids increase (e.g., during fasting, hypocaloric diets, exercise). Although recombinant full-length ANGPTL4 increases plasma TG levels upon intravenous injection, its physiological significance remains unclear [108,134,144].

## 5. TRE: Achieving the Optimal Balance of ANGPTL4 and ANGPTL8?

Figure 6 provides a summary of the concepts discussed to this point, now contextualized by considering the actions of ANGPTL8 and contrasting the fed and fasting states. Food intake robustly induces ANGPTL8 expression, which is restricted to hepatic and adipose tissues. Notably, ANGPTL8 is highly expressed in brown adipose tissue, where its expression is further elevated upon cold exposure. The inhibitory effect of ANGPTL8 on LPL activity is mediated through complex formation with ANGPTL3. Following secretion into the bloodstream, hepatic ANGPTL8 forms an ANGPTL3/8 complex (ratio 3:1), acting via an endocrine mechanism to suppress LPL in oxidative tissues [107,108,109,136,144]. Furthermore, white adipose tissue (WAT)-derived ANGPTL8 interacts with ANGPTL4, forming a complex that counteracts ANGPTL4-mediated inhibition of LPL. Thus, ANGPTL8 indirectly enhances WAT LPL activity. Thus, antibodies targeting the ANGPTL3/8 complex significantly lower triglyceride levels in both mice and humans, preventing the ANGPTL3/8-mediated inhibition of LPL [123,136,141,142,143,144].

Human studies reveal that serum ANGPTL8 concentrations decline physiologically during overnight fasting and increase two hours postprandially. Circulating ANGPTL8, ANGPTL3/8, and ANGPTL4/8 complex levels are elevated by food consumption but decrease with fasting or physical activity [142,143,145,146,147]. Extensive data from human genome-wide association studies and murine loss- and gain-of-function experiments confirm that ANGPTL8 acts as a feeding-induced hepatokine regulating LPL activity via complex formation with ANGPTL3 and ANGPTL4. Associations between ANGPTL8 levels, clinical disorders, and non-metabolic functions, such as inflammation, have also been investigated [112,146,148].

We suggest that significant advancements in this field justify further research into the roles of ANGPTL4 and ANGPTL8 in mediating the positive effects of TRE on TRL dyslipidemia, and their potential to reduce cardiovascular risk. As we have seen, LPL plays a critical role in systemic energy metabolism, prompting ongoing discussion regarding its evolutionary significance. During evolution, malnutrition significantly threatened human survival. According to the thrifty gene hypothesis, evolutionary pressures favored genes that promoted adipose tissue accumulation, enabling individuals to endure periods of famine by storing more fat. ANGPTL8 may represent a thrifty gene, given its primary function in facilitating fat storage postprandially. While this mechanism likely conferred a survival advantage to early humans, in modern contexts, elevated ANGPTL8 activity is associated with an increased risk for metabolic syndrome. Chronic nutritional abundance promotes persistently high ANGPTL8 levels, thereby contributing to excessive adipose deposition (obesity) and elevated triglycerides [112,139,142,146,149]. Conversely, TRE consistently lowers ANGPTL8 levels for a period of 16+ h daily, further substantiating our argument.

Suppressing ANGPTL8 activity could potentially counteract these so-called “thrifty” phenotypes, namely obesity, hypertriglyceridemia, and metabolic syndrome. Animal studies demonstrate that ANGPTL8 deficiency leads to reduced adiposity and serum triglyceride concentrations [141,142,145,146,149]. Similarly, the administration of ANGPTL8 antibodies in mice consistently results in lower fat mass and circulating triglycerides. Additional studies are necessary to establish whether this strategy can mitigate metabolic syndrome in humans with comparable efficacy. As previously outlined, TRE, by enabling a daily window of sixteen hours or more with reduced ANGPTL8 secretion, presents a cost-effective and accessible method to lower overall ANGPTL8 levels.

On the other hand, drugs that target LPL regulators, including ANGPTL3, ANGPTL4, ANGPTL8, ANGPTL3/8, ApoAV, ApoCIII, and ApoCII, are being developed to achieve this goal [124,125,127,128,137,143,147,149,150,151,152,153,154]. These medications tackle different facets of TG metabolism and include mimetic peptides, antisense oligonucleotides, monoclonal antibodies, and small interfering RNAs. It is critical to consider these emerging agents as complimentary therapies that each target distinct aspects of TG metabolism rather than as substitutes for one another. A comprehensive strategy may enhance the management of metabolic syndrome and associated disorders as knowledge of triglyceride metabolism expands and a broader spectrum of LPL regulator-based therapeutics becomes accessible.

Consequently, it is advisable to prioritize further research into the influence of TRE on LPL modulation via ANGPTL4 and ANGPTL8, which provides a natural, accessible, low-cost alternative.

## 6. Chylomicrons vs. VLDL: Role of TRE

In addition to what we highlighted for ANGPTL4, another important mechanism by which TRE reduces atherogenic TRL remnants merits discussion. It has been known for years that the actual larger postprandial increase in TG is not due to chylomicrons (CM), but to VLDL. This may sound paradoxical: the postprandial increase in VLDL levels raises the question of why VLDL concentrations rise when CM are primarily responsible for TG transport during this phase [51,52,55,56,57,58,148]. Specifically, 70–90 g of TG per day (approximately 100 mmol) are transported via CM, while VLDL accounts for 20–30 g per day (about 30 mmol). The elevation in apoB48- (CM) and apoB100-containing VLDL particles contributes to the increased particle number, despite dietary fat carried in CM representing approximately 80% of the postprandial TG elevation [148,155,156]. It is important to note that each CM contains a higher mass of TG per apoB molecule—a marker of particle number—than VLDL. Moreover, CM catabolism is extremely rapid as they are the preferred substrate for LPL. This makes physiological sense, as it provides a quick assimilation of fat after a meal.

**Figure 7:**
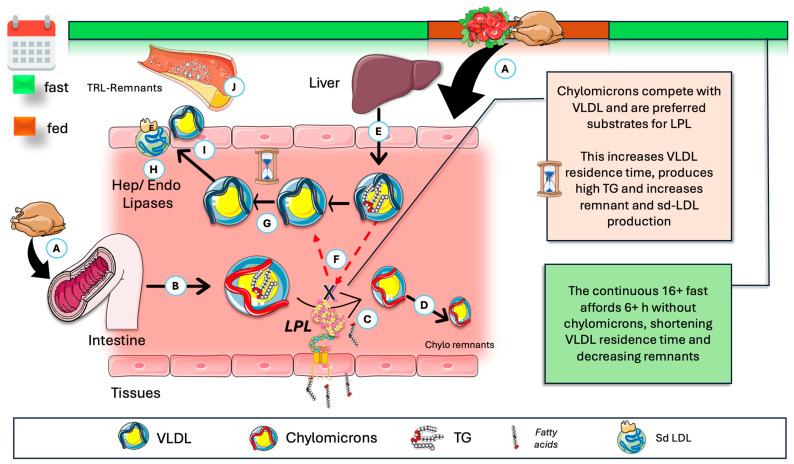
Fasting, via absence of chylomicrons (CM), further optimizes VLDL catabolism and reduces residence time. (**A**) During the postprandial period, CM are produced (**B**) and are acted upon by LPL (**C**) in a very fast catabolism (**D**), leading to remnants taken up by the liver. The liver secretes VLDL continuously (**E**). CM are the preferred substrate, effectively competing for LPL (**F**). This results in (**G**) longer residence times and opportunity for further actions of HL and EL (**H**), favoring the production of remnants and smaller LDL (**I**), which are atherogenic (**J**). Ergo, TRE, by its reduction in the time frame where CM are in circulation, indirectly favors and optimizes VLDL catabolism. CM: chylomicrons; HL: hepatic lipase; EL: endothelial lipase; Sd-LDL: small and dense LDL. The Figure was partly generated using Servier Medical Art, provided by Servier, licensed under a Creative Commons Attribution 3.0 unported license.

A corollary to the above contention is a critical concept with significant pathophysiological implications, which is depicted in Figure 7: the rise in postprandial VLDL may be attributed to competition for the limited availability of lipoprotein lipase (LPL), with larger CM being the preferred substrate [8,148,157,158,159,160,161,162,163]. This competitive interaction explains the strong correlation observed between fasting plasma triglyceride concentrations and the extent of alimentary lipemia following fat consumption. When CM are present, VLDL catabolism is impaired. The frequency and duration of feeding consequently result in intermittent periods of reduced VLDL metabolism by LPL. Referring to Figure 2, VLDL particles with a prolonged residence time become substrates for hepatic lipase (HL) and endothelial lipase, thereby generating small dense LDL and other atherogenic remnants. TRE limits these effects to a much shorter period daily.

Under normal metabolic conditions, the greater size of chylomicron remnants and the relatively lower surface area of apoB48 compared to apoB100 facilitate enhanced apoE binding, leading to increased hepatic uptake and a shorter plasma half-life (minutes for CM versus hours for VLDL). Typically, plasma chylomicron metabolism yields large, lipid-rich, buoyant remnants, whereas VLDL remnants progressively convert to intermediate-density lipoproteins (IDL), which subsequently become low-density lipoproteins LDL [148,157,163].

## 7. Clinical Implementation Guidelines

TRE presents a promising strategy for selected patients; a careful patient selection, deliberate implementation, thorough monitoring, and individualized risk assessment are critical for optimizing outcomes and ensuring safety.

### 7.1. Patient Assessment and Counseling

Conduct a comprehensive health evaluation, including baseline metrics such as weight, body mass index (BMI), blood pressure, and metabolic markers such as fasting glucose and insulin. Assess the patient’s lifestyle—work schedules, social interactions, and sleep habits—to individualize recommendations for the eating window.

Deliver clear, concise information regarding the TRE protocol. Ensure the patient comprehends the rationale for maintaining a consistent eating interval and understands the role of fasting. Distinguish between caloric and non-caloric beverages during fasting periods. Provide educational resources, such as informational sheets and mobile applications for tracking adherence.

Recommend a progressive adjustment to the restricted eating window over several weeks to enhance compliance and minimize transient side effects such as hunger or headaches.

Maintain regular communication with patients, particularly in the early stages, through follow-up visits or telephone consultations to offer support and address any challenges.

Incorporate behavioral interventions, including goal setting, self-monitoring, and facilitation of social support. Practical strategies might include reminders for start and end times of the eating window.

### 7.2. Patient Selection Criteria

#### 7.2.1. Appropriate Candidates for TRE Are as Follows

- Individuals with obesity or metabolic syndrome, where TRE has demonstrated beneficial effects on body composition and cardiometabolic markers.
- Patients with prediabetes or non-insulin-dependent type 2 diabetes, under close medical supervision to ensure safe glycemic control.
- Individuals with cardiovascular risk factors, given evidence of improvements in blood pressure and lipid profiles.
- Individuals seeking straightforward weight management methods without calorie counting.

#### 7.2.2. The Contraindications for TRE Are as Follows

- Pregnant or breastfeeding women, due to increased nutritional requirements.
- Children and adolescents in rapid growth phases, where continuous nutrition is essential.
- Individuals with current or prior eating disorders, as fasting may exacerbate disordered behaviors.
- Patients with type 1 diabetes or insulin-dependent type 2 diabetes, owing to hypoglycemia risk.
- Patients who are underweight or malnourished.
- Patients on medications requiring food intake or those where dosage adjustments are complex; these should be closely monitored by healthcare providers.

### 7.3. Monitoring Parameters

- Monitor body weight, BMI, and body composition to assess progress.
- Track fasting glucose, insulin, and lipid profile parameters (total LDL and HDL cholesterol, triglycerides).
- Regularly assess blood pressure and, when applicable, HbA1c.
- Evaluate consistency of adherence to the prescribed eating window using digital tools or manual records.
- Record and address side effects such as headache, dizziness, fatigue, nausea, or gastrointestinal complaints.
- Periodically review dietary quality to ensure balanced nutrient intake within the eating timeframe.
- Assess psychological well-being, mood, and energy levels, recognizing that fasting may influence these factors variably.
- Monitor sleep duration and quality, as circadian rhythms may impact or be impacted by TRE.

### 7.4. Safety Considerations: High-Risk Populations and Contraindications

Reiterate that TRE is unsuitable for those with eating disorders, pregnant or lactating women, growing children, and individuals at risk of malnutrition.

#### 7.4.1. Medication Interactions

Patients with chronic conditions such as diabetes, hypertension, or cardiovascular disease require careful supervision, with a consideration for potential medication adjustments to mitigate adverse outcomes.

#### 7.4.2. Potential Adverse Effects

Initial adaptation may lead to symptoms such as cefalea, fatigue, or gastrointestinal discomfort, which typically resolve with time. Poor dietary choices during the eating interval can result in inadequate nutrient intake. There is also a risk of compensatory overeating, which may counteract benefits or contribute to negative behavioral patterns.

#### 7.4.3. Cardiovascular and Metabolic Concerns

- A very recent paper [164] suggests caution with narrow eating windows, particularly in populations with pre-existing heart disease or cancer, pending confirmation and further research validation.

## 8. Conclusions

Residual cardiovascular risk remains an issue for patients achieving recommended LDL-C levels, highlighting the need to reassess approaches to atherosclerotic cardiovascular disease. Beyond lipid reduction, factors like inflammation, TRL, lipoprotein(a), and metabolic issues also contribute to RCR. Beyond inflammation treatments targeting the IL-1beta pathway, as described in Section 2.1.1, as well as emerging Lp(a) approaches (Section 2.1.2), triglyceride-lowering therapy aims to reduce circulating TRL remnants rather than just lowering triglycerides. New therapies using antibody and RNA-silencing technologies offer greater specificity and effectiveness compared to older drugs like fibrates, niacin, and omega-3 poly-unsaturated fatty acids (PUFAs), which can lower triglycerides by up to 40% in severe cases. However, studies such as FIELD, ACCORD, and PROMINENT show limited cardiovascular benefits for fibrates, including pemafibrate. While high-dose EPA showed a significant risk reduction in the REDUCE-IT trial, this was not replicated in other studies, possibly due to placebo effects [3,5,12,165,166].

Statins, PCSK9 inhibitors, and ezetimibe are highly effective at reducing LDL-C but only moderately lower triglycerides (5–15%). Current research targets ApoCIII and ANGPTL3, key regulators of lipid metabolism, using antisense oligonucleotides and monoclonal antibodies. These approaches have shown promise in lowering triglyceride levels, particularly in treating rare conditions like familial chylomicronemia syndrome (FCS), while new therapies such as volanosersen [150] significantly reduce triglycerides and may help prevent acute pancreatitis [166,167,168]. Evinacumab, an ANGPTL3 inhibitor, effectively lowers both triglycerides and LDL-C in certain genetic conditions, offering a new treatment option [150]. As the understanding of ASCVD shifts beyond LDL-C and therapies that act on various aspects of the triglyceride metabolism, encompassing mimetic peptides, antisense oligonucleotides, monoclonal antibodies, and small interfering RNAs targeting LPL regulators—such as ANGPTL3, ANGPTL4, ANGPTL8, ANGPTL3/8, ApoAV, ApoCIII, and ApoCII—reach general use, refs. [55,108,125,126,151,166,169,170] dietary interventions such as TRE are increasingly recognized for their potential benefits in metabolic health and weight management. Its basic idea of limiting daily caloric intake to a window of 6 to 8 h makes it arguably the most accessible of Intermittent Fasting approaches, all of which prioritize meal timing over stringent calorie or food quality limits. The advantages of TRE go beyond weight reduction, altering glucose management, blood pressure, and lipid profiles via processes like circadian synchronization and metabolic switching.

Our hypothesis posits that a pivotal mechanism underpinning the cardiovascular and metabolic advantages associated with TRE resides in its influence on the circadian regulation of ANGPTL4 activity within adipose tissue, a process potentially enhanced by diminished ANGPTL8 concentrations. During periods of fasting, ANGPTL4 operates as an autocrine modulator in white adipose tissue, orchestrating the degradation of lipoprotein lipase (LPL) and consequently reducing the availability of active adipose LPL, which is crucial for triglyceride hydrolysis. Moreover, the activation of ANGPTL4 has been demonstrably linked to a reduction in adipose tissue mass. This highlights a dual molecular basis for TRE’s effectiveness at the level of adipocytes: a lower uptake and increased release of fat. We further assert that TRE optimizes intravascular VLDL catabolism efficiency by mitigating lipoprotein lipase’s exposure to chylomicrons, which would otherwise retard VLDL catabolism. This optimized mechanism culminates in a shortened VLDL residence time, thereby restricting the enzymatic actions of hepatic lipase and endothelial lipase and ultimately leading to a reduction in the generation of remnant particles which are, as we have seen, key factors in RCR.

While TRE shows significant promise and is generally well-tolerated, the research field is still developing. Current human studies often have limitations in size and duration, and some findings indicate benefits of small magnitude or null results in certain populations. Future research needs to focus on larger, longer-term randomized controlled trials in diverse populations, with a rigorous methodology to fully elucidate TRE’s role in both disease prevention and treatment. Among the numerous unanswered concerns pertaining to the role of TRE in curbing RCR that will undoubtedly lead to extensive investigation in the years to come, the following come to mind:

- Is our hypothesis correct, or are the proposed mechanisms (namely TRE action on ANGPTL4 and limiting time frame of LPL exposure to CM) simply the result of a better insulin response and kinetics?
- What controls the final integration of the regulatory effects of decreased appetite, increased AMPK activity, and better insulin kinetics during TRE?
- What other nutritional and hormonal factors influence ANGPTL3, ANGPTL4, and ANGPTL8 activity, and what would happen in longer TRE periods?
- Does TRE reduce RCR even when apoCIII levels are elevated?
- What is the final mediator that controls ANGPTL4 expression during TRE?

Until these proposed questions and other queries are answered, and more comprehensive data is available, individuals considering TRE to reduce RCR should prioritize a balanced eating window (6–8 h), maintain a high-quality diet, and always consult with a healthcare professional to ensure its suitability for their specific health needs. We propose that gaining a better understanding of how TRE impacts LPL may lead to breakthroughs capable of reducing the significant RCR observed following LDL-C optimization and potentially lower the reliance on costly (and, in some cases, understudied) emergent ASO, siRNA, and mAb therapies. Lifestyle treatments such as TRE may be critical for improving patient outcomes and managing cardiovascular risk.

## Data Availability

Not applicable.

## Abbreviations

ADF	Alternate-Day Fasting
AgRP	Agouti-related neuropeptide
AMPK	AMP activated kinase
ANGPTL4	Angiopoietin-like protein 4
ASCVD	Atherosclerotic cardiovascular disease
BMI	Body mass index
CVD	Cardiovascular disease
CART	Cocaine–amphetamine-regulated transcript
CETP	Cholesteryl-ester transfer protein
CM	Chylomicrons
CII	ApoCII
CIII	ApoCIII
E	ApoE
ERK	Extracellular-signal regulated kinase
FA	Fatty acids
FOXO	Forkhead box transcription factors
GLP-1	Glucagon-like peptide-1
GPIHBP1	Glycosylphosphatidylinositol-anchored high-density lipoprotein binding protein 1
HDL	High-density lipoproteins
HDL-C	High-density lipoprotein cholesterol
HSPG	Heparan sulfate proteoglycans
hsCRP	High-sensitivity C-reactive protein
HSL	Hormone-sensitive lipase
IGF-1	Insulin-like growth factor-1
IL-1 β	Interleukin-1β
IDL	Intermediate-density lipoproteins
IF	Intermittent Fasting
IR	Insulin resistance
LDL-C	Low-density lipoprotein cholesterol
LDL-R	LDL receptor
Lp(a)	Lipoprotein (a)
LPL	Lipoprotein lipase
LRP-1	LDL-receptor like protein 1
mTORc	Mammalian target of rapamycin c
MetS	Metabolic syndrome
NPY	Neuropeptide Y
PCSK3	Proprotein convertase subtilisin/kexin type 3.
PPAR	Peroxisome proliferator–activated receptors
PUFA	Poly-unsaturated fatty acids
POMC	Pro-opium melanocortin
PCSK9	Proprotein convertase subtilisin/kexin type 9
RCR	Residual cardiovascular risk
sd-LDL	Small–dense LDL
SIRT1	Sirtuin 1
siRNA	Small interfering RNA
SGLT-2	Sodium-glucose co-transporter-2
SCN	Suprachiasmatic nucleus
TRE	Time-restricted eating
TG	Triglyceride
TRL	Triglyceride-rich lipoprotein
VLDL	Very low-density lipoproteins.
WAT	White adipose tissue
